# Disseminated *Geosmithia argillacea* Infection in a Patient with Ph-Positive Acute Lymphoblastic Leukemia. Case Report and Literature Review

**DOI:** 10.3390/jof7090778

**Published:** 2021-09-19

**Authors:** Antonio Giordano, Francesca Di Landro, Elena De Carolis, Marianna Criscuolo, Giulia Dragonetti, Luana Fianchi, Livio Pagano

**Affiliations:** 1 Department of Hematology, Fondazione Policlinico Universitario Agostino Gemelli—IRCCS, 00168 Roma, Italy; francescadilandro92@gmail.com (F.D.L.); marianna.criscuolo@policlinicogemelli.it (M.C.); dragonettigiulia@gmail.com (G.D.); luana.fianchi@policlinicogemelli.it (L.F.); 2 Department of Microbiology, Fondazione Policlinico Universitario Agostino Gemelli—IRCCS, 00168 Roma, Italy; elena.decarolis@policlinicogemelli.it; 3 Institute of Hematology, Faculty of Medicine and Surgery, Università Cattolica del Sacro Cuore, 00168 Roma, Italy

**Keywords:** acute lymphoblastic leukemia, mold

## Abstract

Invasive fungal infection (IFI) remains the major complication in patients with either acute leukemia, allogeneic stem cell transplantation setting, or both, especially regarding pulmonary localization. We report an experience of a 74-year-old Caucasian male with a Philadelphia-positive (BCR-ABL p190) Common B-acute lymphoblastic leukemia (ALL) who developed a pulmonary infection due to *Geosmithia argillacea*. Furthermore, we describe the management of this complication and the results of microbiological tests useful to guide the treatment. All cases reported show failure of voriconazole treatment. In the majority of cases a good susceptibility to posaconazole has been reported, which seems to have a good clinical impact; however, only L-AmB shows a clinical effect to produce quick clinical improvement and so it should be a drug of choice. A literature revision shows that only a few papers have thus far described this infection, at present only one case was reported in a hematological setting like a gastrointestinal graft versus host disease in an allogeneic HSCT recipient. The severity of clinical conditions in hematological malignancy settings requires improving the management of this emerging invasive fungal infection. Indeed, a molecular diagnostic approach with a tight laboratory collaboration and targeted therapy should become the gold standard.

## 1. Introduction

Invasive fungal infection (IFI) remains the major complication in patients with either acute leukemia, allogeneic stem cell transplantation setting, or both, especially with regard to pulmonary localization. The percentage of patients who develop IFIs has increased dramatically in recent decades [1].

Furthermore, these patients have an increased risk of developing IFI due to the presence of central venous catheters, prolonged neutropenia, and high dose of steroid treatment with or without chemotherapy. Stratifying risk for invasive fungal infection is the logical first step in identifying patients who would most likely benefit from antifungal prophylaxis, more intensive monitoring, or early treatment [2].

The incidence of proven or probable mold and yeast infections can reach 24% among patients with leukemia [3]. Reported mortality from candidiasis or aspergillosis ranges from 40 to 50% [4], and mortality from fusariosis or zygomycosis is 70% or more [5,6].

The use of posaconazole in prophylaxis has reduced the incidence of IFIs in acute myeloid leukemia (AML) [7], thus this may be a useful addition to the standard of care for patients with AML or myelodysplastic syndromes who are undergoing induction chemotherapy. However, drug interactions prevent its use in cases of acute lymphoblastic leukemia (ALL) which are therefore prone to developing IFIs sustained by both molds and yeasts.

Fluconazole prophylaxis is also used in other patient populations with neutropenia, although fewer data support its efficacy in these patients. Fluconazole has an acceptable adverse-event profile but lacks efficacy against filamentous fungi, which have become increasingly frequent causes of infection in patients with neutropenia [8]. Itraconazole has a wider spectrum of activity than fluconazole, including activity against aspergillus species [9].

Several studies, most of which published over the last five years, analyzed the rate of IFI in patients with acute lymphoblastic leukemia (ALL), showing an overall incidence of mold infections ranging between 3 and 12% [10].

*Geosmithia argillacea* can colonize to form a fungal ball, manifesting as hemoptysis, especially in patients without underlying diseases (such as cystic fibrosis) that predispose them to respiratory pathogens and in immunocompromised patients (such as post-transplantation). The aim of this paper is to describe the presentation of an emerging invasive infection in order to better understand diagnostic approach and treatment.

## 2. Case Report

We report an experience of a 74-year-old Caucasian male with a Philadelphia-positive (BCR-ABL p190) Common B-ALL who was treated according to risk-adapted protocol with a high dose of steroid and tyrosine kinase inhibitors (imatinib) along with central nervous system prophylaxis. Five years earlier the patient underwent excision of right inferior pulmonary lobe due to lung cancer, with no evidence of disease recurrence.

The patient started antiviral and antimicrobial prophylaxis with acyclovir, trimethoprim-sulfamethoxazole, and fluconazole. After 60 days from the beginning of tyrosine kinase treatment, the patient showed cutaneous rash and dyspnea, thus imatinib was stopped assuming drug intolerance. Following the appearance of productive cough, multidrug-resistant *Acinetobacter baumanii* and *Pseudomonas aeruginosa*, as well as fungal infection by *Aspergillus fumigatus* and *Aspergillus terreus,* was isolated from sputum analysis. In the absence of fever or other systemic symptoms, the patient started outpatient treatment with both antibiotic and antifungal treatment with oral voriconazole (200 mg bid).

The simultaneous flow cytometric analysis showed an increase of minimal disease residue, thus the patient was switched to the second generation of tyrosine kinase inhibitor (dasatinib) and continued steroid treatment.

**Figure 1:**
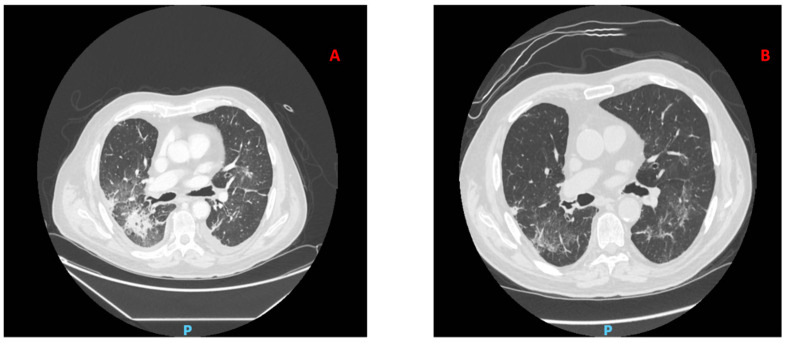
Radiological images (**A**) CT scan pre-therapy; (**B**) CT scan 21 days after therapy with L-AmB.

After two weeks from the beginning of voriconazole he was hospitalized for fever and respiratory distress: a computed tomography scan (CT-scan) of the lungs revealed extensive areas of parenchymal consolidation with areas of hyperdensity of the adjacent pulmonary parenchyma with a “ground glass” appearance (Figure 1A). The patient underwent bronchoscopy exam and broncho-alveolar lavage (BAL). *Proteus mirabilis*, *Enterococcus faecalis*, and *Candida glabrata* were isolated; in addition, BAL galactomannan fluid assay was positive, whereas serum galactomannan value was always negative. He started targeted antimicrobial therapy and antifungal treatment was switched to isavuconazole. However, performance status and oxygen requirements worsened during hospitalization and hemoptysis appeared. Growth of an unspecified mold was discovered from lavage fluid, so the patient was switched to liposomal amphotericin B (L-AmB) (3 mg/Kg per day). There was a progressive improvement in clinical conditions and dyspnea.

**Figure 2:**
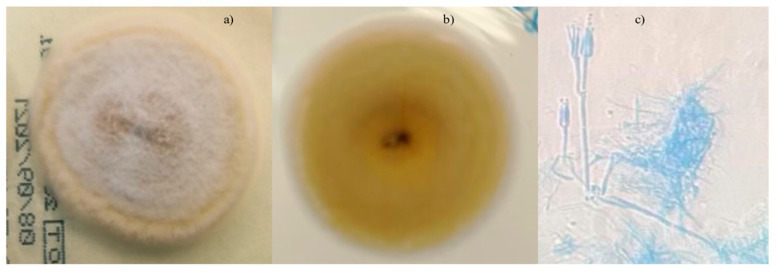
Microbiological assay images. (**a**) Front creamy colored; (**b**) reverse brown surface of *Geosmithia argillacea* mycelium; (**c**) microscopic lactophenol cotton blue stain; 400×.

After two weeks from the onset, the cultures were identified as the mold, *Geosmithia argillacea* (Figure 2).

It can potentially be misidentified as other genera due to morphological similarities. The mold was retrieved from the bronchoalveolar fluid cultured at 30 °C on Saboraud dextrose agar plates (Vacutest Kima S.r.l, Padova, Italy) according to our fungal growth protocol. Microscopic examination of the cultures revealed hyaline, septate conidiophores bearing cylindrical phialides and conidia.

After mycelial DNA extraction, PCR was performed using Hotstart Taq Master Mix Kit (Qiagen, Hilden, Germany) and fungal β-tubulin primers Bt2a(5-GGTAACCAAATCGGTGCTGCTTTC) and Bt2b(5-ACCCTCAGTGTAGTGACCCTTGGC-3) [11].

Species identification was performed by database searching with the BLAST sequence analysis tool (http://www.ncbi.nlm.nih.gov/BLAST/ accessed on 3 April 2021), 100% identity was reported for *Geosmithia argillacea* with accession number HQ686280. Antifungal susceptibility test (AFST) was performed by broth microdilution according to CLSI methods for filamentous fungi (Sensititre YeastOne ITAMYUCC, Thermo Scientific).

**Figure 3:**
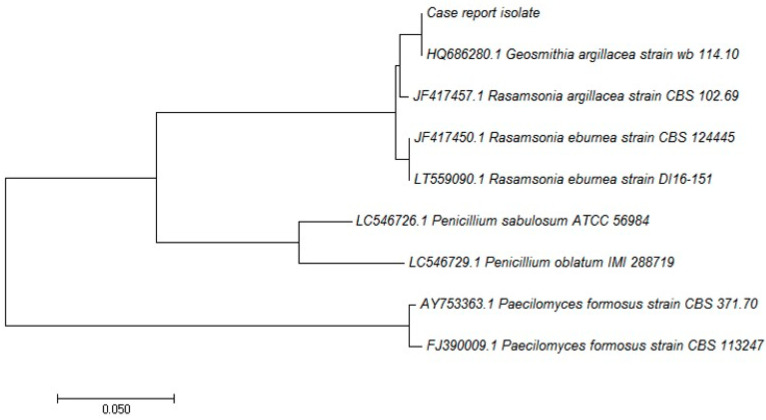
Phylogenetic tree.

Based on a β-tubulin gene sequence alignment including the case report isolate, closed related sequences, and sequences from microscopically similar fungi (*Paenicillium* and *Paecilomyces*), a phylogenetic tree (Figure 3) was constructed by MEGA7 (Molecular Evolutionary Genetics Analysis) (Kumar ref) using the neighbor-joining method [12].

Unrooted neighbor-joining phylogenetic tree based on beta-tubulin gene sequence of *Geosmithia argillacea* and microscopically similar fungi. The evolutionary distances were computed using the maximum composite likelihood method; the units of the number of base substitutions per site are indicated in the scale bar.

Susceptibility tests revealed a high in vitro minimum inhibition concentration against voriconazole (>8 mg/L), isavuconazole (>8 mg/L), and fluconazole (>256 mg/L), but lower minimum inhibition concentration to amphotericin B (1 mg/L), posaconazole (0.125 mg/L), itraconazole (0.5 mg/L), and caspofungin (0.008–0.06 mg/L).

After three weeks of antifungal treatment with L-AmB (cumulative dose 3780 mg), a new lung CT scan showed a marked improvement of pulmonary picture (Figure 1B), and the patient was discharged from the hospital in good enough clinical condition and without any clinical signs of active infection.

In light of results regarding susceptibility, the test patient continued at-home prophylaxis with oral posaconazole.

Unfortunately, two months later he showed underlying malignancy progression and died due to leukemia without signs of active fungal infection.

## 3. Discussion

*Geosmithia argillacea* is an anamorph of *Talaromyces eburneus*, a thermophilic filamentous fungus which has a phenotype similar to that of the *Penicillium* or *Paecilomyces* species, except for the creamy-white colonies not compatible with those of *Penicillium spp*. and cylindrical conidia. This fungus is part of the *Rasamsonia* species complex which has recently been proposed thanks to the reclassification by molecular methods of *Talaromyces* and *Geosmithia* species. Due to the high similarity with *Paenicillium* and *Paecilomyces* genera, the real incidence of *Geosmithia* spp. could be underestimated and would need further awareness especially in the presence of suggestive microscopic morphology in hematological patients. Molecular methods should be the key for accurate identification at the species level and along with the antifungal susceptibility test are very important in infection management. Although the galactomannan positive test is a non-specific reaction, together with the macroscopic aspect of the creamy colored colonies it could be considered as a warning bell to perform deeper analysis.

With improvement in diagnostic techniques, particularly DNA sequencing, species within the *Rasamsonia argillacea* species complex have been reported with increasing frequency as a cause of invasive disease amongst vulnerable populations.

A literature review shows that only few papers until now described this infection. The first case of a *G. argillacea* infection was reported in 2009 in a German shepherd dog [13]. The pathogenic potential of *G. argillacea* in humans was first reported after it was detected in the sputum of patients with cystic fibrosis in 2010 [14] (Table 1) and now it is considered one of the rare respiratory pathogens associated with the pathophysiology of cystic fibrosis [15].

It has been suggested that an immunocompromised state following lung transplantation might make a patient susceptible to a severe *G. argillacea* infection [15]. In 2011, various cases of invasive mycosis or disseminated diseases caused by *G. argillacea* were reported in patients with chronic granulomatous disease [16,19], and in 2013 a case report paper described the infection in a patient with tuberculosis. An existing pulmonary cavity that was created during a previous lobectomy provided space for the fungal ball to grow. Although there was no evidence of immuno-deficiency providing vulnerability to infection, we cannot exclude the possibility of the presence of a predisposing factor that promoted either the colonization, infection, or in combination, by *G. argillacea*. Increased awareness among clinicians and microbiologists is necessary for them to fully comprehend the implications of a *G*. *argillacea* infection and understand the pathophysiology of this fungus [17].

At present only one case was reported in a hematological setting, such as a gastrointestinal graft versus host disease in an allogeneic HSCT recipient [18].

The outcome of this complication is poor, and all cases reported show failure of voriconazole treatment in most cases with only a good susceptibility to posaconazole, which seems to have a good clinical impact. In fact, this mold shows resistance to latest-generation azoles including isavuconazole. On the contrary, L-AmB shows a clinical effect of producing quick clinical improvement and as such it should be a drug of choice; and regarding our experience, we also achieved a good improvement of clinical conditions with L-AmB administration.

The severity of clinical status in hematological malignancy settings requires improving the management of this emerging invasive fungal infection. Indeed, a molecular diagnostic approach with a tight laboratory collaboration and targeted therapy should become the gold standard.

This invasive infection was not reported until now in the setting of hematological malignancies treated with conventional induction chemotherapy or in refractory disease, and it was probably sometimes mistaken for other fungi. So, it is very important to correctly identify the kind of infection and subtype of molds in order to guide the target treatment and radically change prognosis in hematological malignancy patients.

More cases are needed to determine the long-term prognosis and the best form of therapy for this disseminated fungal infection. Due to similar clinical, imaging, and histopathologic features to disseminated *Aspergillus* and *Penicillium* species, *Geosmithia argillacea* should be considered a differential diagnosis.

## Figures and Tables

**Table 1:** Cases of *Geosmithia argillacea* reported in literature.

References	Setting	N. Patients	Signs and Symptoms	Diagnosis	Antifungal Susceptibilities	Treatment	Outcome
Giraud et al. J Clin Microbiol, 2010 [14]	Cystic Fibrosis	17	Hemoptysis Cough Fever	Lung secretions	L-Amb, Posaconazole, Caspofungin	posaconazole	100% alive
Barton et al. J Clin Microbiol, 2010 [15]	Cystic Fibrosis	Not reported	Cough Sputum	Sputum samples	L-Amb, Itraconazole, Caspofungin, Posaconazole	Not Reported	Not Reported
De Ravin et al. Clin Infect Dis, 2011 [16]	Chronic Granulomatous Disease	7	Cough Fever	Lung secretions	L-Amb, Posaconazole, Micafungin	Voriconazole	Failure in 6 of 7 patients
Sohn et al. Ann Lab Med, 2013 [17]	Tuberculosis	1	Hemoptysis Fever	BAL	NT	Not reported	Not reported
Valentin et al. BMT, 2012 [18]	GVHD in HSCT	1	Hemoptysis Diarrhea	BAL	L-Amb, Posaconazole, Caspofungin	Voriconazole	Failure

BAL: bronchoalveolar lavage; GVHD: graft versus host disease; HSCT: hematopoietic stem cell transplantation.

## Data Availability

Not applicable.
